# A combined experimental and modeling study to evaluate pH-dependent sorption of polar and non-polar compounds to polyethylene and polystyrene microplastics

**DOI:** 10.1186/s12302-018-0155-z

**Published:** 2018-08-14

**Authors:** Sven Seidensticker, Peter Grathwohl, Jonas Lamprecht, Christiane Zarfl

**Affiliations:** Center for Applied Geoscience, Eberhard-Karls Universität Tübingen, Tübingen, Germany

**Keywords:** Microplastics, Sorption, Equilibrium distribution

## Abstract

**Background:**

The contamination of aquatic ecosystems with both anthropogenic pollutants and particles in particular (microscopic) plastic debris items is of emerging concern. Since plastic particles can accumulate contaminants and potentially facilitate their transport, it is important to properly investigate sorption mechanisms. This is especially required for a large variety of chemicals that can be charged under environmental conditions and for which interactions with particles may hence go beyond mere partitioning.

**Results:**

In this study, sorption experiments with two types of microplastic particles (polyethylene and polystyrene) and 19 different contaminants (pesticides, pharmaceuticals, and personal care products) were performed at three different pH values. We could show that sorption to plastic particles is stronger for hydrophobic compounds and that neutral species usually contribute more to the overall sorption. Bulk partitioning coefficients were in the same order of magnitude for polyethylene and polystyrene. Furthermore, our results confirm that partition coefficients for polar compounds can only be accurately determined if the solid-to-liquid ratio in batch experiments is more than 6–7 orders of magnitude higher than any plastic concentration detected in the environment. Consequently, only a minor fraction of pollutants in water bodies is associated with microplastics.

**Conclusions:**

Although neutral species primarily dominate the overall sorption, hydrophobic entities of ionic species cannot be neglected for some compounds. Notwithstanding, our results show that since microplastic concentrations as currently observed in the environment are very low, they are only a relevant sorbent for strongly hydrophobic but not for polar compounds.

**Electronic supplementary material:**

The online version of this article (10.1186/s12302-018-0155-z) contains supplementary material, which is available to authorized users.

## Introduction and background

Chemical pollution is of emerging concern and has even been considered to be a planetary boundary threat [6, 32]. In addition, contamination with anthropogenic litter such as microplastics raises public concern [3, 15, 27]. Since, in freshwaters, pollutants distribute between the freely dissolved water phase and natural but also anthropogenic particles, microplastics may be an additional vector for spreading pollutants [20]. Therefore, one important aspect is to assess the impact of particle-bound contaminants. Since transport of particles and associated contaminants, i.e., for particle-facilitated transport, is different compared to purely dissolved chemicals [2], sorption/desorption kinetics, and thus, particle properties such as density, shape, material, and size are decisive. Sorbed contaminants are less available for biodegradation and may, therefore, be transported further than chemicals freely available in the aqueous phase that are more easily prone to transformation processes [1, 10]. Furthermore, determined partition coefficients can serve for an improved understanding of sorption processes and, hence, a deepened risk assessment of sorbed contaminants. For compounds with slow desorption kinetics, i.e., with high partition coefficients [36], microplastics may act as transport vectors, whereas they act as passive samplers and reflect the ambient concentration of the organic pollutants in the environment as soon as sorption equilibrium is reached. In both cases, partition coefficients are crucial to calculate (i) characteristic times for contaminant release and (ii) the ambient concentration (e.g., in the water) at the sampling location. Kinetics and impact of partition coefficients are thoroughly discussed in Seidensticker et al. [36]. In freshwater systems, one major path for microplastics to enter rivers is via the effluent of wastewater treatment plants where large numbers of particles have been observed [28, 29]. In such effluents, however, also high concentrations of micropollutants such as pharmaceuticals and pesticides occur [12, 13, 23, 35]. Therefore, plastic particles might especially act as a sorbent and potential transporter for frequently occurring wastewater contaminants, and are, hence, a factor that needs to be considered if the environmental fate of micropollutants is examined.

Depending on their physico-chemical properties, many pollutants dissociate under certain pH conditions, and hence, their fate and behavior in the environment is strongly influenced by changes in the pH [5, 19]. Among these ionizing chemicals are pesticides, flame retardants, but also pharmaceuticals and further household chemicals like detergents. From studies with natural particles and sediments, it is known that sorption interaction mostly takes place between sorbents and neutral species and that charged compounds sorb only little or not at all [11, 19]. Unlike many natural particles, microplastics can be charged electrostatically [37, 41]. Therefore, the uptake of ionizable substances theoretically might not only determined by mere partitioning between microplastic particles and the neutral species but also by possible ionic bounds. Sorption interactions between charged species and different types of microplastics have not been extensively studied so far. Thus, the aim of our study was to clarify the sorption behavior to microplastics in freshwater under varying pH of five neutral substances (as control) and a set of 14 selected ionizable compounds including pesticides and insecticides, but also pharmaceuticals, detergents, and flame retardants that represent trace pollutants emitted via the wastewater treatment plant. Pristine polyethylene and polystyrene particles were used, since, in the wastewater canalization system, “young” particles occur and enter the WWTP where they get in contact with various (emerging) contaminants such as pharmaceuticals and personal care products which we used in this study. Both plastic types are among the most abundant in WWTP (as, e.g., reported in Mintenig et al. [28] and Murphy et al. [29]).

## Methods

### Chemicals

In total, 19 different chemicals were tested for their sorption interactions with different microplastics. These chemicals include seven bases with dissociation constants (pK_a_) ranging from 1.09 to 8.37, eight acids covering pK_a_ values of 3.13–13.9, and four neutral substances. Details on the physico-chemical properties of these compounds are listed in Table 1.

**Table 1:** Physico-chemical properties of the investigated substances

Compound	CAS#	Molecular weight (g mol^−1^)	WS_sub_ (mol L^−1^)	log *K*_OW_	pK_a_	Acid–base reaction
Atrazine	1912-24-9	215.69	5.43 × 10^−3^	2.61	1.60	Base
Benzotriazole	95-14-7	119.13	1.05	1.44	8.37	Base
Caffeine	58-08-2	194.19	1.65 × 10^1^	0.07		Neutral
Carbamazepine	298-46-4	236.28	3.10 × 10^−3^	2.45	13.9	Acid
Carbendazim	10605-21-7	191.19	9.27 × 10^−2^	1.52	4.29	Base
DEET	134-62-3	191.28	Liquid	2.18		Neutral
Diazinon	333-41-5	304.35	1.31 × 10^−3^	3.81	2.60	Base
Diclofenac	15,307-86-5	296.15	1.87 × 10^−4^	4.51	3.99	Acid
Ibuprofen	15687-27-1	206.29	3.69 × 10^−4^	3.97	4.45	Acid
MCPA	94-74-6	200.62	3.13 × 10^−2^	3.25	3.13	Acid
Mecoprop	7085-19-0	214.65	1.62 × 10^−2^	3.20	3.78	Acid
4-Nonylphenol	104-40-5	220.36	4.78 × 10^−5^	5.76	10.7	Acid
Phenanthrene	85-01-8	178.24	3.95 × 10^−5^	4.46		Neutral
Propiconazole	60207-90-1	342.22	Liquid	3.72	1.09	Base
Tris(2-chloroisopropyl)-phosphate (TCPP)	13674-84-5	327.57	Liquid	2.59		Neutral
Tebuconazole	107534-96-3	307.83	7.78 × 10^−4^	3.70	1.76	Base
Terbutryn	886-50-0	241.36	7.15 × 10^−4^	3.74	4.30	Base
Torasemide	56211-40-6	348.42	2.10 × 10^−2^	3.37	6.68	Acid
Triclosan	3380-34-5	289.55	7.55 × 10^−5^	4.76	7.90	Acid

Properties are either taken from EPISuite (molecular weight and log *K*_OW_ of neutral species) or the PubChem database (pK_a_). Subcooled liquid solubilities (WS) were estimated based on melting points according to Kan and Tomson [18] and Liu et al. [24]

All chemicals except phenanthrene were purchased from LGC standards (Wesel, Germany). The latter was purchased from Sigma-Aldrich Supelco (Bellefonte, PA, USA).

Polyethylene (PE) and polystyrene (PS) were used as representative types of microplastics, and were purchased from Azelis (trade name Gotalene 120, Moers, Germany) and Goodfellow Cambridge Ltd. (Huntingdon, UK), respectively. Sizes of polyethylene and polystyrene microparticles were given by the supplier (uniform size distribution with mean sizes of 260 and 250 µm, respectively), and confirmed by visual inspection under SEM (see images in the Additional file 1). Particles with comparable sizes were chosen to better compare sorption mechanisms and to exclude huge differences due to size effects. Furthermore, *N*_2_-BET surface areas were measured and revealed that PE is non-porous, while PS is mesoporous with an average pore size of ~ 195 Å. According to Pascall et al. [31], glass transition temperatures *T*_g_ of polyethylene and polystyrene are in the range of − 120 and 100 °C, respectively.

### Batch experiments

To study equilibrium partitioning of charged and non-charged compounds, we performed batch experiments with ultrapure water (electric conductance of 0.057 µS cm^−1^) and microplastic particles at three different pH levels (4, 7, and 10) and the mix of selected substances. For all compounds, the initial concentrations in the water phase were around 5 µg L^−1^ except for phenanthrene and nonylphenol. Due their high hydrophobicity, these two substances were expected to sorb very strongly. Therefore, the initial concentrations were 50 and 30 µg L^−1^, respectively, to avoid aqueous concentrations below the detection limit. The initial concentrations of all substances were below 1% of their water solubility to avoid competitive sorption. The batches were spiked from an aqueous contaminant solution to avoid co-solvent effects of organic solvents. Either formic acid or ammoniac was used to adjust the pH in the batches. For preparing the neutral solution, 0.02 M Na_2_PO_4_ (Rotifair, Carl Roth) was used. For each pH, the solutions were prepared in one glass vessel before they were distributed into the single batches. During the experiments, frequent pH measurements were performed to control the stability. All experiments were prepared in ultrapure water and performed in amber glass bottles to avoid biodegradation and photo-oxidation. Blanks were included to confirm that neither biodegradation nor sorption to glass walls or seals, etc. takes place. The liquid-to-solid ratio in the batches was 0.001 kg L^−1^, namely 100 mg of microplastics in 100 mL of solution. Batch experiments for each pH were performed in triplicates and samples of 2 × 1 mL were taken at the beginning (*t* = 0) to quantify the actual initial concentration. Further samples were taken after 2, 4, 7, and 11 (only for PS) days to measure the overall partitioning of the substances and to ensure that equilibrium was reached within the batches. Samples were taken from completely independent batches, and all samples were considered to take outliers into account, as well. For detailed studies on kinetics and conformation of fast equilibration, see Seidensticker et al. [36]. The bottles were constantly shaken on a horizontal shaker with a rotational speed of 150 rpm and kept in a dark room tempered to 20 °C. The sampling procedure ensured that the liquid-to-solid ratio changed less than 10%. Thus, this minor change was neglected in the subsequent data analysis, since it is in the range of the analytical error. As discussed below, only partition coefficients larger than 50 L kg^−1^ can be reliably determined, since, at larger liquid-to-solid ratios, the measurement errors escalate. Therefore, all data resulting in smaller partition coefficients are not reported. Furthermore, the coefficients of variation (CV) have been calculated as the standard deviation normalized to the mean values of the replicates and given in %. CV values > 100% were as well a criterion for exclusion.

### Chemical analysis

Phenanthrene, nonylphenol, and TCPP were quantified via GC–MS. For analysis, an Agilent 6890N GC coupled to an Agilent 7973 inert MS was used. For separation, a J + W Scientific DB-5MS (30 m length, 0.025 mm ID, and 0.25 µm film thickness) capillary column was used. The device was operated in a pulsed splitless mode with a Helium flow of 0.7 mL min^−1^. Samples were taken as described above and internal standards (Phenanthrene-D_10_ and 4-*n*-Nonylphenol-D_8_) were added. Subsequently, the samples were extracted with 400 µL of cyclohexane, shaken overnight, and measured.

The other 16 substances were quantified with LC–MS/MS and samples were directly injected after gravitational phase separation. Since the LC system is equipped with a pre-column filter, remaining particles would not be able to enter the column and to produce false-positive results. For quantification, a calibration curve with nine different concentration levels from 0.025 to 10 µg L^−1^ was generated. For analysis, an Agilent 1290 infinity LC coupled to an Agilent 6490 Triple Quadrupole was used. Separation was performed with an Agilent InfinityLab C18 poroshell column (length 100 mm, 2.1 µm ID). For elution, water (with 0.1% acetic acid and 0.01 mM ammonium acetate) and acetonitrile (ACN, with 0.1% acetic acid) were used. The gradient elution looks as follows (with percentage of ACN): start with 2%, stepwise increase to 80% until 17 min, 100% until 23 min, then again 2% until 32 min. For the quantification of the samples from the batch experiments with either triclosan or diclofenac, two isocratic methods with 57% ACN or 70% ACN were used, respectively. For these measurements, specific calibration curves were generated, as well (concentration range from 0.5 to 250 µg L^−1^ with seven calibration levels). Substances were ionized with an ESI source operated either in positive mode or negative mode. Details on the ionization mode, mass transitions, and other analytical characteristics are reported in the Additional file 1.

### Model-based data analysis

Linear partitioning of a substance between two phases, here water and a type of microplastics, is given as the equilibrium partition coefficient *K*_P_ (L kg^−1^), i.e., the concentration ratio of the sorbed [*C*_p_ in (µg kg^−1^)] and dissolved [*C*_w_ in (µg L^−1^)] fraction:

1 $${K_{\text{P}}} = \;\frac{{{c_{\text{P}}}}}{{{c_{\text{W}}}}}{\text{\;in\;equilibrium}} .$$

For ionizable compounds, the pH-dependent partition coefficient *D*_P_ can be calculated as follows:

2 $${D_{\text{P}}} = \;{K_{\text{P,n}}}{f_{\text{n}}} + {K_{\text{P,i}}}{f_{\text{i}}},$$

where *f*_n_ and *f*_i_ are the fractions of the neutral and ionized species, and *K*_P,n_ and *K*_P,i_ are the species-specific partition coefficients for the neutral and the ionized species, respectively. *f*_n_ and *f*_i_ were calculated from the known pK_a_ and pH values according to the rearranged Henderson–Hasselbalch equation, while the species-specific partition coefficients were deduced from fitting the calculated *D*_P_ value to the experimentally determined *D*_P_ values. At each pH and for each substance, *D*_P_ was calculated from 9 or 12 measured aqueous concentrations for PE and PS, respectively. No measurement results were excluded. For the fitting procedure, a MATLAB Code (Version R2017b) was used. Within this code, a non-linear least-squares solver was used to calculate *K*_P,n_ and *K*_P,i_ from fitting Eq. 2 to measured *D*_P_ values. This procedure allows calculating a theoretical *D*_P_ for each substance over the full pH range. To assess the uncertainty of the determination of partition coefficients, a simple error evaluation was considered as follows:

3 $$\frac{{{K_{\text{P,c}}} - {K_{\text{P,m}}}}}{{{K_{\text{P,c}}}}} = \;\frac{{\varepsilon + \frac{{{V_{\text{W}}}}}{{{m_{\text{P}}}{K_{\text{P,c}}}}}\varepsilon }}{{\left( {1 + \varepsilon } \right)}} = \;\frac{\varepsilon }{1 + \varepsilon }\left( {1 + \frac{{{V_{\text{W}}}}}{{{m_{\text{P}}}{K_{\text{P,c}}}}}} \right),$$

with *K*_P,c_ and *K*_P,m_ as the calculated and measured partition coefficients, respectively, and *V*_W_, *m*_P_, and *ε* denote the volume of water, the mass of particles, and the uncertainty of the measurement (e.g., a standard deviation).

### Aqueous pollutant concentrations under two different microplastic scenarios

Experimental results on the overall sorption coefficients of the investigated substances at three pH values were used to calculate two scenarios with different microplastic concentrations. In both scenarios, organic carbon (values for *K*_OC_ were taken from US EPA EPISuite 4.1 and apply for the so-called “normal” soil organic matter) was present as a natural sorbent which can compete for the sorbates and is able to act as a vector, as well. To compare the results from our experiments with an environmental relevant setting, two different liquid-to-solid ratios (LSR) concerning the amount of plastics were chosen. To match our experimental conditions, in Scenario I, a LSR of 10^3^ L kg^−1^ and to reflect particle concentrations closer to current environmental conditions in Scenario II, an LSR of 10^10^ L kg^−1^ (comparable to conditions at the effluent of WWTPs, according to particle concentrations recorded by Mintenig et al. [28]) were chosen. The mass of organic carbon was set to 10 mg, i.e., a concentration of 10^−5^ kg L^−1^. The dissolved fraction ${f_{\text{diss}}}$ in the water, i.e., the ratio between the equilibrium and the initial concentration, may be easily calculated as follows:

4 $${f_{\text{diss}}} = \frac{1}{{1 + {K_{\text{OC}}}\;{m_{\text{OC}}}/{V_{\text{W}}} + {K_{\text{P}}}\;{m_{\text{P}}}/{V_{\text{W}}}}}.$$

Here, *V*_W_, *m*_OC_, and *m*_P_ denote the volume of water, the mass of organic carbon, and the mass of plastic particles, respectively. Each scenario was calculated over a range of *K*_OC_ and *K*_P_ values and for the three different pH values that were used in the experiments. For the investigated substances, *K*_P_ was quantified with the particle-water partition coefficient that was experimentally determined.

## Results and discussion

### Equilibrium sorption to PE

**Fig. 1:**
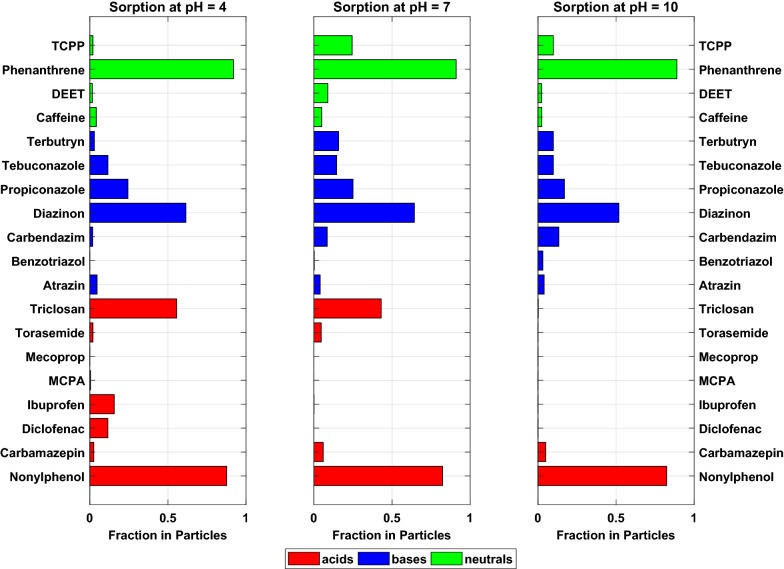
Sorbed fractions of the investigated contaminants to polyethylene at different pH levels. The bars are colored according to the acid/base properties of the substances as indicated in the legend. For substances for which a negative *D*_P_ has been determined, the sorbed percentage was set to zero

Sorption to PE is, in general, strongly dependent on the substance properties and is mostly driven by partitioning. Polymer properties as, e.g., density [30], branching of polymer chains and crystallinity [8] may, as well, influence sorptive interactions. Sorption of non-polar compounds is stronger than sorption of polar compounds, and sorption of charged species is weaker than sorption of neutral species (Fig. 1). Fluctuations of sorbed percentages will be discussed below. Detailed sorption plots of every substance and the respective agreement with the model can be found in the Additional file 1. In general, the model could be fitted to the measured overall partition coefficients quite well. The derived *K*_PE,n_ and *K*_PE,i_ are listed in Table 2; to secure reliability, exact values were only reported if *K*_PE_ were > 50 L kg^−1^ and/or CV were < 100%. Even though it is expected that the species-specific partition coefficient of the ions is zero or close to zero, for some compounds, the species-specific partition coefficients indicate that the charged species contribute to sorption, as well. For these cases, the *K*_PE,i_’s difference from zero is greater for weakly sorbing compounds. These results will be discussed in more detail below.

**Table 2:** *K*_PE,n_ and *K*_PE,i_ values derived from the model fit (Eq. 2) to measured *D*_P_ values and compared to the log *K*_OW_

Compound	log *K*_OW_	*K*_PE,n_ (L kg^−1^)	*K*_PE,i_ (L kg^−1^)	% variation coefficients of *D*_P_		
				pH 4	pH 7	pH 10
Atrazine	2.61	< 5 × 10^1^	< 5 × 10^1^	49.3	60.1	54.7
Benzotriazole	1.44	< 5 × 10^1^	< 5 × 10^1^	185.3	69.2	38.8
Caffeine	0.07	< 5 × 10^1^	n.a. (no ionic species)	69.4	63.1	157.0
Carbamazepine	2.45	6.7 × 10^1^	< 5 × 10^1^	129.2	59.3	60.6
Carbendazim	1.52	< 5 × 10^1^	< 5 × 10^1^	161.8	54.2	57.3
DEET	2.18	5.2 × 10^1^	n.a.	122.0	44.3	100.8
Diazinon	3.81	1.75 × 10^3^	< 5 × 10^1^	39.0	75.8	14.7
Diclofenac	4.51	1.5 × 10^2^	< 5 × 10^1^	57.9	61.6	133.6
Ibuprofen	3.97	2.6 × 10^2^	1.9 × 10^2^	35.5	59.4	81.0
MCPA	3.25	8.8 × 10^3^	< 5 × 10^1^	74.2	163.3	13,130.3
Mecoprop	3.20	< 5 × 10^1^	< 5 × 10^1^	146.7	522.4	139.9
4-Nonylphenol	5.76	6.0 × 10^3^	7.2 × 10^2^	13.5	13.6	12.4
Phenanthrene	4.46	9.9 × 10^3^	n.a.	8.7	12.4	28.3
Propiconazole	3.72	3.4 × 10^2^	< 5 × 10^1^	45.0	52.0	26.2
TCPP	2.59	2.2 × 10^2^	n.a.	77.5	84.8	24.6
Tebuconazole	3.70	1.4 × 10^2^	< 5 × 10^1^	48.0	75.0	35.4
Terbutryn	3.74	6.2 × 10^1^	6.3 × 10^1^	31.8	53.8	18.1
Torasemide	3.37	1.3 × 10^2^	< 5 × 10^1^	210.6	34.1	34.0
Triclosan	4.76	1.1 × 10^3^	< 5 × 10^1^	27.8	38.2	203.1

*K* values below 50 L kg^−1^ are not reported due to too large uncertainties

For some substances, sorption did not significantly decrease with increasing share of ionized species, indicating that structural features as, e.g., the hydrophobic neutral tail of the surfactant-like nonylphenol are responsible for sorptive interactions.

### Equilibrium sorption to PS

**Fig. 2:**
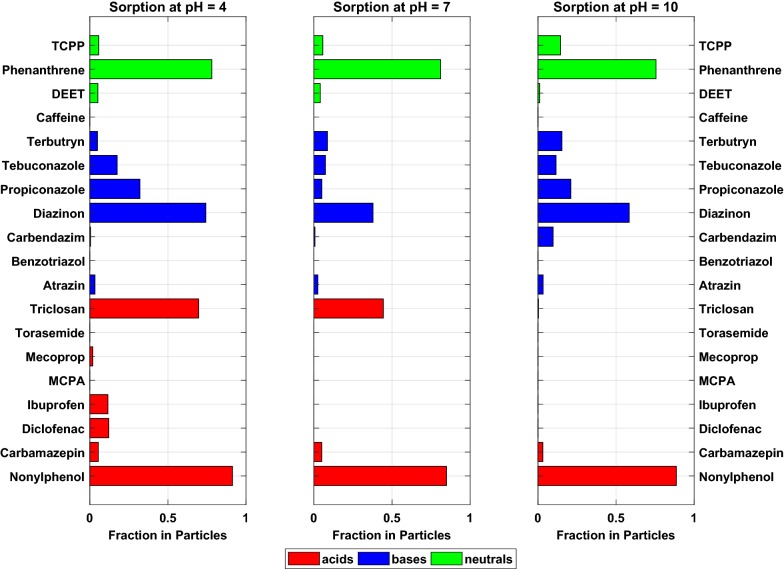
Sorbed fractions of the investigated contaminants to polystyrene at different pH levels. The bars are colored according to the acid/base properties of the substances as indicated in the legend. For substances for which a negative *D*_P_ has been determined, the sorbed percentage was set to zero

Sorption to PS was stronger than sorption to PE for most of the investigated substances and driven by both partitioning and adsorption (e.g., a pore-filling mechanism confirmed by the non-linear sorption isotherms (as provided in the Additional file 1) which is in agreement with the findings of other authors [16, 39]. Nevertheless, sorption coefficients for both plastic types are within the same order of magnitude. Furthermore, in analogy to the case for PE, sorption to PS was driven by hydrophobicity as well and substances that sorbed strongly to PE also sorbed strongly to PS (Fig. 2). Detailed plots of measured and modeled *D*_P_ for all substances can be found in the Additional file 1. Again, the model could be fitted to measured overall partition coefficients well and, again, the model fits were better for stronger sorbing compounds. Based on the deduced *K*_PS,n_ and *K*_PS,i_, sorption of neutral species to PS is stronger than to PE, whereas sorption of the ionic species is weaker for most of the substances (Tables 2 and 3).

**Table 3:** *K*_PS,n_ and *K*_PS,i_ values derived from the model fit (Eq. 2) to measured *D*_P_ values and compared to the log *K*_OW_

Compound	log *K*_OW_	*K*_PS,n_ (L kg^−1^)	*K*_PS,i_ (L kg^−1^)	% variation coefficients of *D*_P_		
				pH 4	pH 7	pH 10
Atrazine	2.61	< 5 × 10^1^	< 5 × 10^1^	244.2	335.9	257.3
Benzotriazole	1.44	< 5 × 10^1^	< 5 × 10^1^	444.7	88.4	213.7
Caffeine	0.07	< 5 × 10^1^	n.a. (no ionic species)	1785.7	428.3	1199.7
Carbamazepine	2.45	< 5 × 10^1^	< 5 × 10^1^	172.2	175.6	283.2
Carbendazim	1.52	< 5 × 10^1^	< 5 × 10^1^	471.1	598.5	117.5
DEET	2.18	< 5 × 10^1^	n.a.	177.1	240.7	500.2
Diazinon	3.81	2.15 × 10^3^	2.09 × 10^3^	93.6	82.5	53.5
Diclofenac	4.51	2.70 × 10^2^	< 5 × 10^1^	93.0	214.2	6101.7
Ibuprofen	3.97	2.05 × 10^2^	< 5 × 10^1^	45.9	184.0	161.0
MCPA	3.25	< 5 × 10^1^	< 5 × 10^1^	699.7	1564.9	196.7
Mecoprop	3.20	< 5 × 10^1^	< 5 × 10^1^	138.6	609.1	2206.8
4-Nonylphenol	5.76	9.18 × 10^3^	3.74 × 10^3^	18.7	49.8	31.2
Phenanthrene	4.46	7.21 × 10^3^	n.a.	29.8	21.5	40.4
Propiconazole	3.72	1.17 × 10^2^	< 5 × 10^1^	44.5	169.0	48.9
TCPP	2.59	1.06 × 10^2^	n.a.	42.5	61.5	30.6
Tebuconazole	3.70	9.89 × 10^1^	< 5 × 10^1^	57.3	97.0	85.7
Terbutryn	3.74	< 5 × 10^1^	< 5 × 10^1^	158.7	166.8	90.2
Torasemide	3.37	< 5 × 10^1^	< 5 × 10^1^	2502.6	95.4	70.5
Triclosan	4.76	5.12 × 10^3^	< 5 × 10^1^	95.6	123.4	241.9

*K* values below 50 L kg^−1^ are not reported due to too large uncertainties

Coefficients of variation increase with increasing share of ionic species, thus decreasing sorption which leads to the conclusion that for weakly sorbing ionic species the error escalates. Differences between sorption to PE and PS can most likely be explained due to the non-linearity of sorption to PS. Consideration of the differences between ambient concentrations and water solubility is crucial if non-linear sorption mechanisms are investigated. Studies performed, e.g., by Hüffer and Hofmann [16] and Lee et al. [21] determined higher partition coefficients for PS, whereas other studies by Pascall et al. [31] indicate stronger sorption to PE. In general, there is a lack of experiments comparing sorption to different types of microplastics. Experiments performed in our own lab showed that sorption of a polycyclic aromatic hydrocarbon (phenanthrene) and two heterocyclic compounds to PE and PS was stronger for PE and revealed a slight non-linearity of sorption isotherms to PS (reported in the Additional file 1). Therefore, we conclude that sorption to PE is driven by partitioning, i.e., absorption, whereas sorption to PS may be driven by both adsorption and subsequent pore-filling mechanisms which are confirmed by the non-linear sorption isotherms. According to the high glass transition temperature of PS, the free volume within the polymeric matrix is low, and hence, adsorption is favored in comparison to absorption [31]. As there are many producers of plastics using different ingredients, the differences between the same types of plastics can be as manifold as the number of manufacturers. Hence, the outcome of such sorption experiments can be different depending on the material supplier and comparability is, in general, difficult.

### Sorption of the ionic species

As expected, the species-specific partition coefficients for most substances were higher for the neutral species; the ionic species of some substances showed some significant sorption, as well. This occurred especially for more hydrophobic substances such as nonylphenol and triclosan, whereas, for weakly sorbing compounds, species-specific partitioning coefficients of the ionic species were at least one order of magnitude smaller. Whereas predictions solely based on the log *K*_OW_ which is the classic parameter for estimating hydrophobicity fail to predict accumulation of ionic species [9], there is some evidence that polar species can accumulate within organisms as well and play an important role in bioaccumulation in fat [7, 14]. In addition, carbon nanotubes can sorb ionic liquids [40].

### Impact of particle concentration

**Fig. 3:**
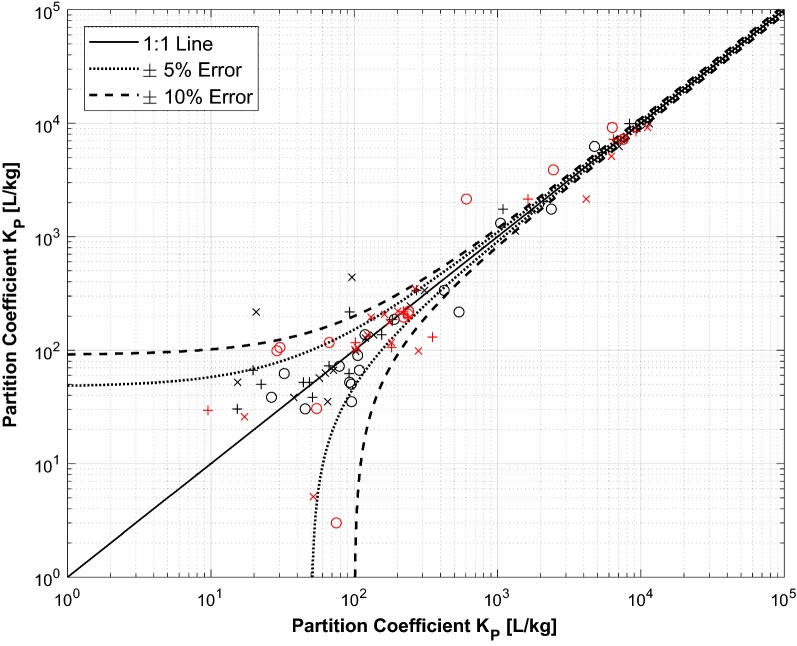
Uncertainty of partition coefficients calculated according to Eq. 3. Uncertainties were determined with standard analytical errors of *ε* = ± 5% (dotted lines) and of *ε* = ± 10% (dashed lines). The differences between the measured *D*_P_ (*x*-axis) and the calculated *D*_P_ (*y*-axis) are displayed by the black and red symbols for PE and PS, respectively. Crosses, circles, and pluses indicate *D*_P_ values determined at pH 4, pH 7, and pH 10, respectively

The best practice to sensitively measure partition coefficients is to choose a liquid-to-solid ratio (LSR) in the same range or lower as the prospective partition coefficient that should be determined. This allows determination of the partition coefficient with a sufficiently low uncertainty. In our experiments, the LSR was 10^3^ L kg^−1^, and thus, only partition coefficients greater than 10^3^ L kg^−1^ or slightly smaller (> 50 L kg^−1^) can be determined with small errors. As it can be seen in Fig. 3, the partition coefficients determined for polar and weakly sorbing compounds are subject to greater uncertainty, and the deviation between the calculated and the measured partition coefficients increases with decreasing sorption independent of the pH. Therefore, the partition coefficients determined for polar and weakly sorbing compounds are subject to greater uncertainty which is reflected in the variation coefficients.

An LSR of 10^3^ L kg^−1^ as chosen in our batch experiments represents microplastics concentrations that are orders of magnitude larger than the ones that were detected in the environment where LSRs are usually > 10^9^ L kg^−1^ [20, 28, 34]. Thus, batch setups focusing on elucidating a specific process detail, like the species-specific sorption coefficients in this study, do not reflect environmental relevant conditions, in particular when considering that plastic debris is just a very minor fraction compared to all natural particles that environmental contaminants can partition to. In addition, for most of the substances investigated within this study, the determined *D*_P_s were below the order of 10^1^ to 10^2^ L kg^−1^, i.e., the lower the particle concentration, the smaller is the mass flux of the substance into the solid phase and, with this, the resulting highly uncertain measurements of *K*_P_. Therefore, adsorption efficiencies reported in the literature (e.g., 60 and 70% for PFC sorption to PE and PS; [25] are only possible under very low (and thus unrealistic) LSRs.

**Fig. 4:**
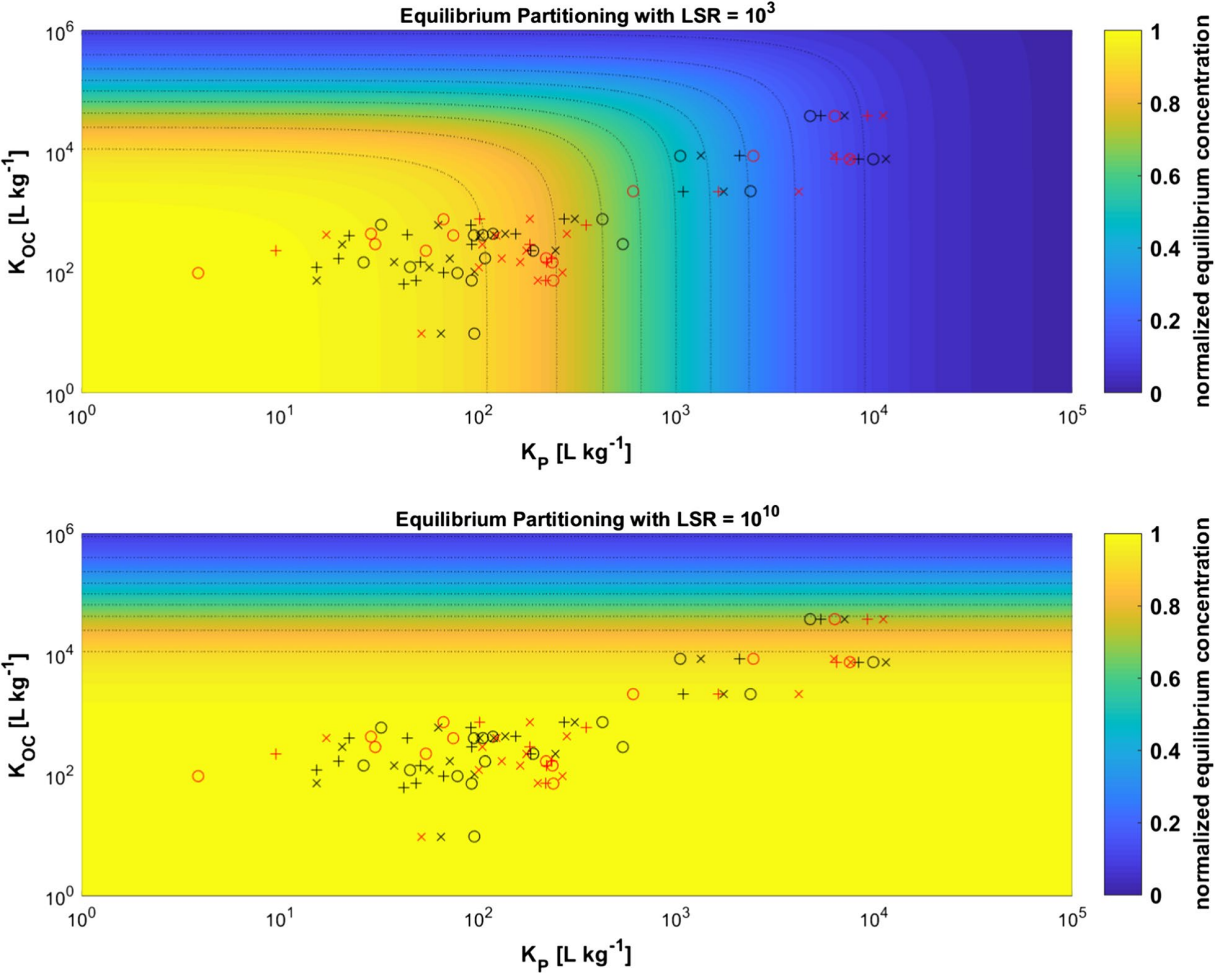
Equilibrium distribution map for a freshwater system with natural particles (organic carbon) and microplastic particles under two different concentration scenarios. Experimental conditions (plastic LSR = 10^3^ L kg^−1^, Scenario I) are shown in the top panel and environmental conditions (plastic LSR = 10^10^ L kg^−1^, Scenario II) in the bottom panel. Crosses, circles, and pluses show the aqueous equilibrium concentrations at pH 4, pH 7, and pH 10, respectively, in µg L^−1^ calculated with the experimentally determined *D*_P_ for PE (black symbols) and for PS (red symbols) and the investigated substances. For both cases, a constant concentration of 10^−5^ kg L^−1^ OC was assumed. *K*_OC_ values were estimated using EPISuite 4.1

This is even more true under environmental conditions as there are more sorbing phases such as black carbon or dissolved organic matter available which take up contaminants as well and partly even much stronger than polymers [4]. Calculations on equilibrium distribution in a freshwater system containing natural sorbents (organic carbon) and microplastic particles show that microplastics are only relevant if their concentration in water is much higher than the concentration of other organic carbon containing phases (Scenario I, Fig. 4, top). However, at environmental relevant concentrations of microplastics, the effect of natural organic particles likely prevails. If *K*_P_ is smaller than the LSR, the distribution of the tested substances shifts and almost all compounds would predominantly be available in the freely aqueous phase (Scenario II, Fig. 4, bottom). Thus, our findings for ionizable compounds also support arguments which state that microplastic particles are not substantial vectors for contaminants in terms of substance mass transported due to their low environmental concentrations [20, 26]. In particular, this is true if considered that at very low, but environmental relevant concentration, e.g., for phenanthrene, field-measured *K*_d_ values for partitioning to suspended sediment particles lead to much larger *K*_OC_ values [33] than estimated from the EPISuite database due to non-linear sorption. Thus, the particles’ sorption capacity may be even larger as assumed in our model calculations. Even though particle properties and sorption interactions may change under environmental conditions in particular due to aging [17], studying sorption to rather pristine particles is highly relevant, since the alteration through aging can only be investigated if sorption processes to pristine particles are known.

## Conclusion and outlook

To assess the potential effects of microplastics and associated contaminants on ecosystems it is important to properly evaluate the particle–pollutant interactions, especially since this determines their bioavailability which is yet not well understood. Hydro- and geochemical parameters as well as contact with biota may change particle characteristics, e.g., their surface charge and texture, and may procure aggregation [22, 38]. Thus, it is essential to analyze whether sorption interactions occur which are going beyond mere partitioning as investigated here. At least for pristine plastic particles, we could show that partitioning is still the main sorption mechanism and sorption of charged species at least with the current environmental microplastic concentrations in freshwaters is irrelevant.

## Additional file

**Additional file 1.** Additional tables and figures.
