# Supplementary material for: A combined experimental and modeling study to evaluate pH-dependent sorption of polar and non-polar compounds to polyethylene and polystyrene microplastics
Source: Environ Sci Eur. 2018 Aug 14;30(1):30. doi: 10.1186/s12302-018-0155-z (PMC6096972; doi:10.1186/s12302-018-0155-z)
Supplement: Supplementary file 1 — Additional file 1. Additional tables and figures. [file 12302_2018_155_MOESM1_ESM.pdf]

Microplastics as contaminant vector: A combined experimental and modeling study to evaluate pH-dependent sorption of polar and non-polar compounds

Sven Seidensticker, Peter Grathwohl, Jonas Lamprecht, Christiane Zarfl

Center for Applied Geoscience, University of Tübingen

Supporting Information

16 pages including

6 Tables
8 Figures

S1 Calculations

To calculate the fractions of neutral and charged species Eq. (S1) and Eq. (S2) were used for acids and bases, respectively.

$$f_{n,a} = \frac{1}{1 + 10^{pH - pK_a}} \tag{S1}$$

$$f_{n,b} = \frac{1}{1 + 10^{pK_a - pH}} \tag{S2}$$

The used pK_a values are listed in Table 1 in the main manuscript.

For all substances the pH-dependent partition coefficient D_P was calculated for each pH. As explained in the main manuscript a *MATLAB* Code using a nonlinear least-square solver was operated to estimate the partition coefficients $K_{P,n}$ and $K_{P,i}$ for the neutral and the ionic species, respectively for both types of plastic particles. Subsequently the theoretical $D_{P,calc}$ were calculated for each pH using Eq. (S3).

$$D_{P,calc} = f_n K_{P,n} + (1 - f_n) K_{P,i} \tag{S3}$$

To compare each of the measured D_Ps with the theoretical $D_{P,calc}$, for each pH, each compound, and the two different types of microplastics error calculations were performed as specified in the manuscript.

S2 Particle Properties

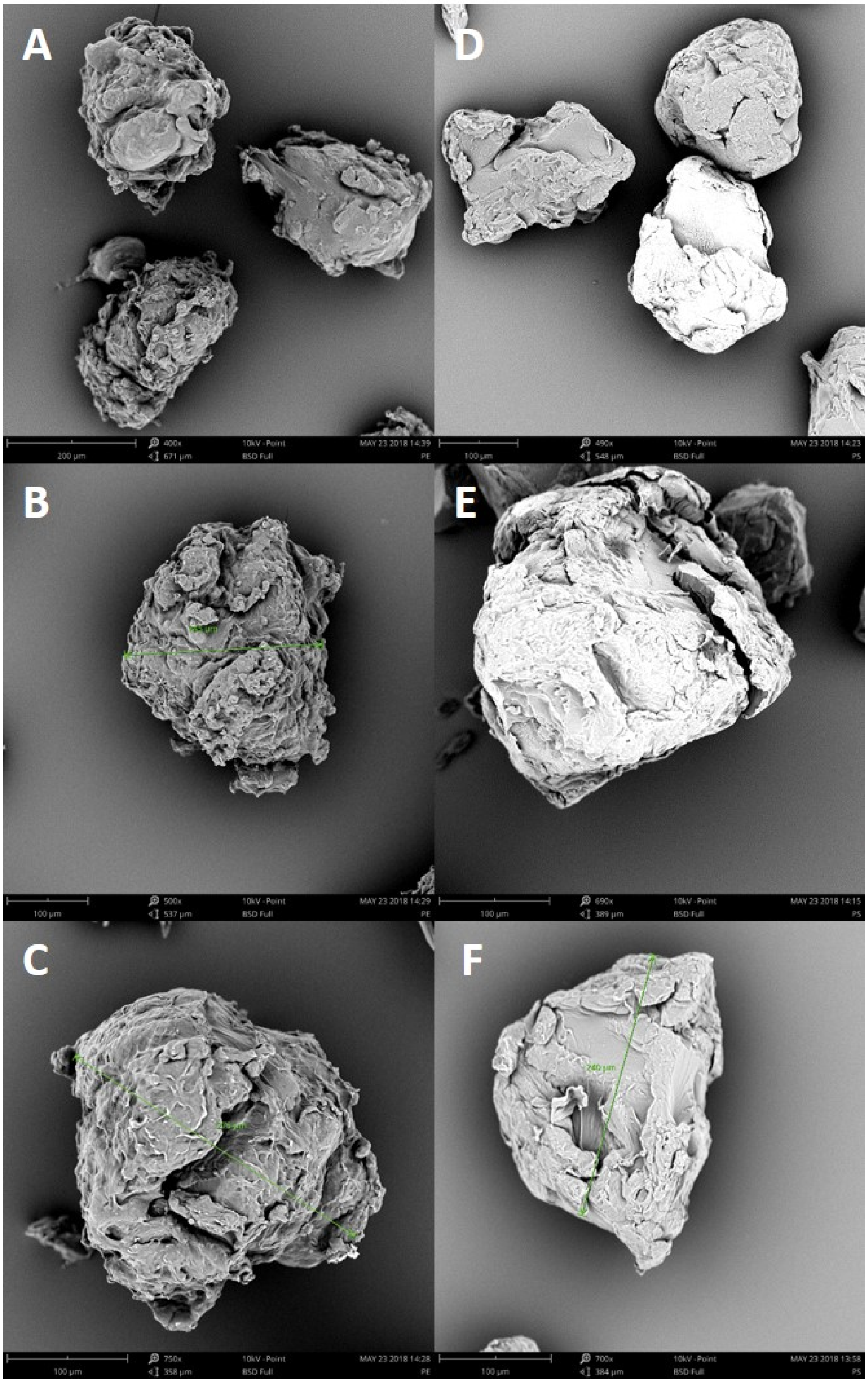

Figure S1: SEM images of used PE (left panel, A-C) and PS (right panel, D-F) particles.

S3 Results for Polyethylene Particles

The measured partition coefficients between the chosen contaminants and polyethylene are listed in Tables S1, S2, and S3 for pHs of 4, 7, and 10, respectively. Comparison between estimated and measured partition coefficients are shown in Figures S2, S3, and S4 for acids, bases, and neutrals, respectively.

Table S1: Measured D_P and calulated $D_{P,calc}$ for sorption of investigated compounds to polyethylene at pH = 4.

Substance	Measured Partition coefficient D_P	Calculated Partition coefficient $D_{P,calc}$
Nonylphenol	7063	6248
Carbamazepin	72	67
Diclofenac	102	98
Ibuprofen	246	246
MCPA	96	438
Mecoprop	<50	-133
Torasemide	120	128
Triclosan	1330	1123
Atrazin	<50	38
Benzotriazol	<50	-9
Carbendazim	57	57
Diazinon	1741	1706
Propiconazole	309	335
Tebuconazole	137	137
Terbutryn	63	63
Caffeine	65	35
DEET	<50	52
Phenanthrene	11451	9909
TCPP	<50	218

Table S2: Measured D_P and calulated $D_{P,calc}$ for sorption of investigated compounds to polyethylene at pH = 7.

Substance	Measured Partition coefficient D_P	Calculated Partition coefficient $D_{P,calc}$
Nonylphenol	4755	6246
Carbamazepin	109	67
Diclofenac	95	50
Ibuprofen	190	187
MCPA	<50	-224
Mecoprop	79	72
Torasemide	106	90
Triclosan	1051	1313
Atrazin	<50	38
Benzotriazol	<50	-5
Carbendazim	<50	30
Diazinon	2376	1750
Propiconazole	425	336
Tebuconazole	119	137
Terbutryn	<50	62
Caffeine	96	35
DEET	93	52
Phenanthrene	9921	9909
TCPP	539	218

Table S3: Measured D_P and calulated $D_{P,calc}$ for sorption of investigated compounds to polyethylene at pH = 10.

Substance	Measured Partition coefficient D_P	Calculated Partition coefficient $D_{P,calc}$
Nonylphenol	5353	5514
Carbamazepin	$<$50	67
Diclofenac	$<$50	50
Ibuprofen	184	187
MCPA	$<$50	-225
Mecoprop	67	73
Torasemide	$<$50	52
Triclosan	2089	2034
Atrazin	51	38
Benzotriazol	$<$50	-42
Carbendazim	$<$50	30
Diazinon	1091	1750
Propiconazole	272	336
Tebuconazole	155	137
Terbutryn	92	62
Caffeine	$<$50	35
DEET	$<$50	52
Phenanthrene	8353	9909
TCPP	93	218

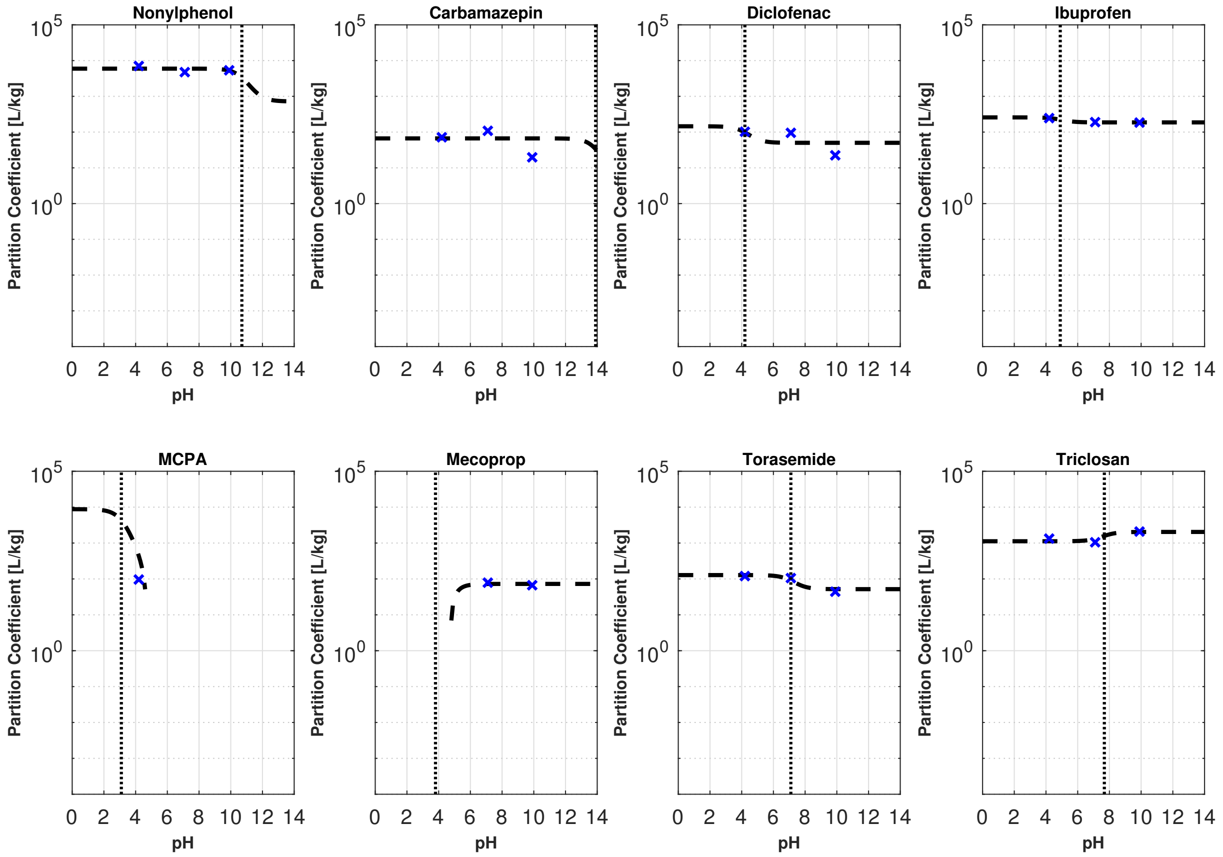

Figure S2: Comparison of measured D_P and theoretical partitioning for sorption of acids to polyethylene illustrated by the blue crosses and dashed lines, respectively. The vertical dotted lines indicate the pK_a values. Due to the log-scale of the y-axis only positive values can be displayed.

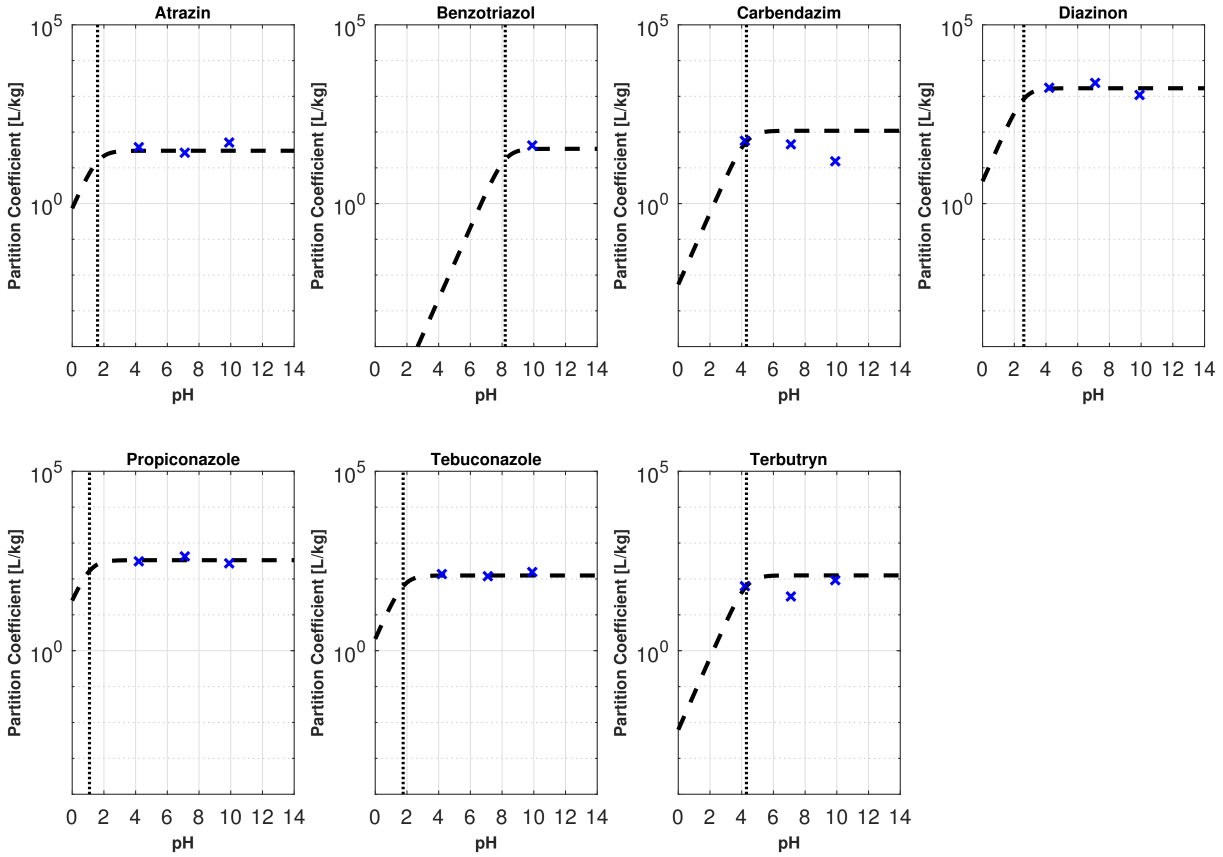

Figure S3: Comparison of measured D_P and theoretical partitioning for for sorption of bases to polyethylene illustrated by the blue crosses and dashed lines, respectively. The vertical dotted lines indicate the pK_a values. Due to the log-scale of the y-axis only positive values can be displayed.

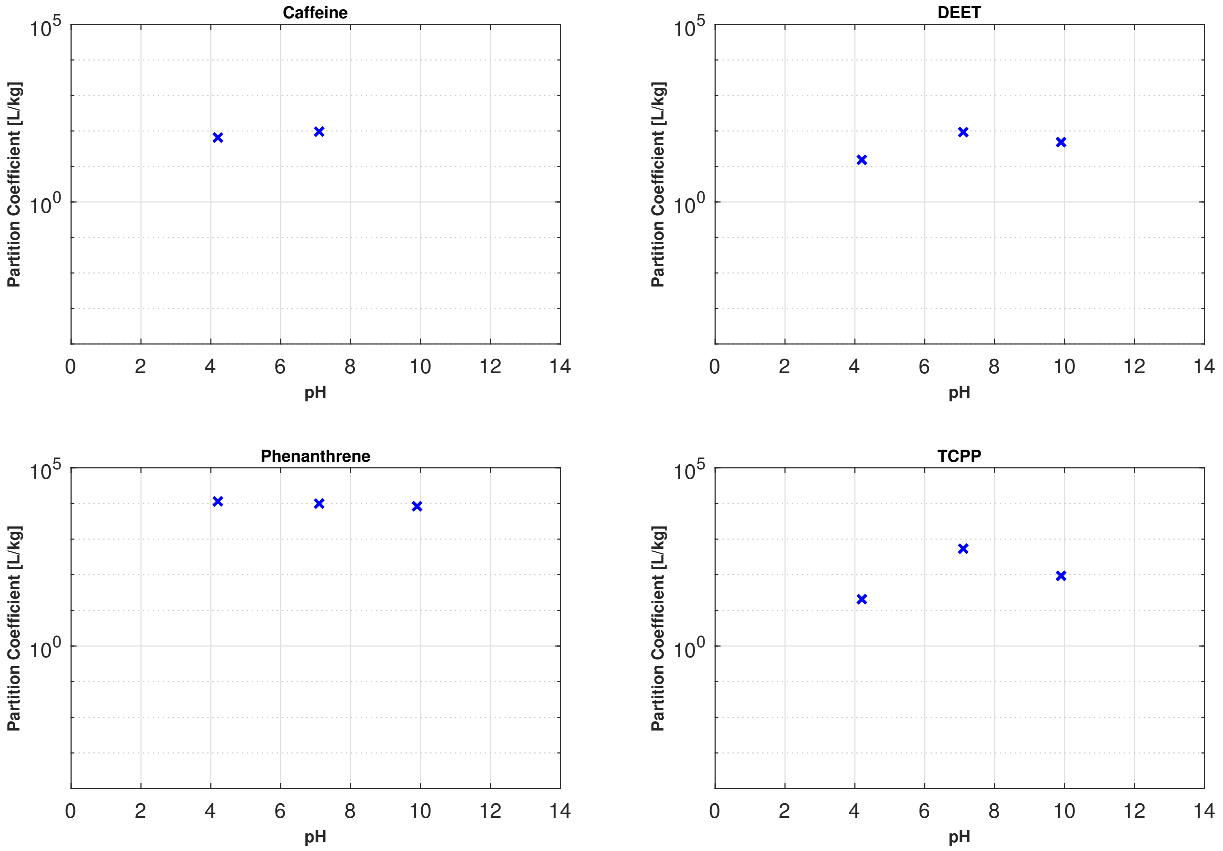

Figure S4: Measured D_P for sorption of neutrals to polyethylene illustrated by the blue crosses. Due to the log-scale of the y-axis only positive values can be displayed.

S4 Results for Polystyrene Particles

The measured partition coefficients between the chosen contaminants and polystyrene are listed in Tables S4, S5, and S6 for pHs of 4, 7, and 10, respectively. Comparison between estimated and measured partition coefficients are shown in Figures S5, S6, and S7 for acids, bases, and neutrals, respectively

Table S4: Measured D_P and calulated $D_{P,calc}$ for sorption of investigated compounds to polystyrene at pH = 4.

Substance	Measured Partition coefficient D_P	Calculated Partition coefficient $D_{P,calc}$
Nonylphenol	11149	9182
Carbamazepin	131	196
Diclofenac	124	137
Ibuprofen	176	176
MCPA	<50	4193
Mecoprop	266	348
Torasemide	<50	26
Triclosan	6243	5114
Atrazin	163	207
Benzotriazol	<50	-19
Carbendazim	101	101
Diazinon	4162	2147
Propiconazole	182	117
Tebuconazole	281	99
Terbutryn	<50	1
Caffeine	52	5
DEET	201	221
Phenanthrene	7623	7212
TCPP	105	106

Table S5: Measured D_P and calulated $D_{P,calc}$ for sorption of investigated compounds to polystyrene at pH = 7.

Substance	Measured Partition coefficient D_P	Calculated Partition coefficient $D_{P,calc}$
Nonylphenol	6330	9180
Carbamazepin	221	196
Diclofenac	75	3
Ibuprofen	55	31
MCPA	<50	-2217
Mecoprop	<50	-26
Torasemide	<50	-24
Triclosan	2456	3885
Atrazin	238	208
Benzotriazol	<50	-23
Carbendazim	<50	-16
Diazinon	607	2148
Propiconazole	67	117
Tebuconazole	<50	99
Terbutryn	<50	130
Caffeine	<50	5
DEET	240	221
Phenanthrene	7549	7212
TCPP	<50	106

Table S6: Measured D_P and calulated $D_{P,calc}$ for sorption of investigated compounds to polystyrene at pH = 10.

Substance	Measured Partition coefficient D_P	Calculated Partition coefficient $D_{P,calc}$
Nonylphenol	9273	8438
Carbamazepin	236	196
Diclofenac	<50	3
Ibuprofen	<50	30
MCPA	<50	-2226
Mecoprop	<50	-26
Torasemide	<50	-73
Triclosan	<50	-766
Atrazin	222	208
Benzotriazol	<50	-82
Carbendazim	<50	-16
Diazinon	1631	2148
Propiconazole	102	117
Tebuconazole	<50	99
Terbutryn	351	131
Caffeine	<50	5
DEET	221	221
Phenanthrene	6465	7212
TCPP	182	106

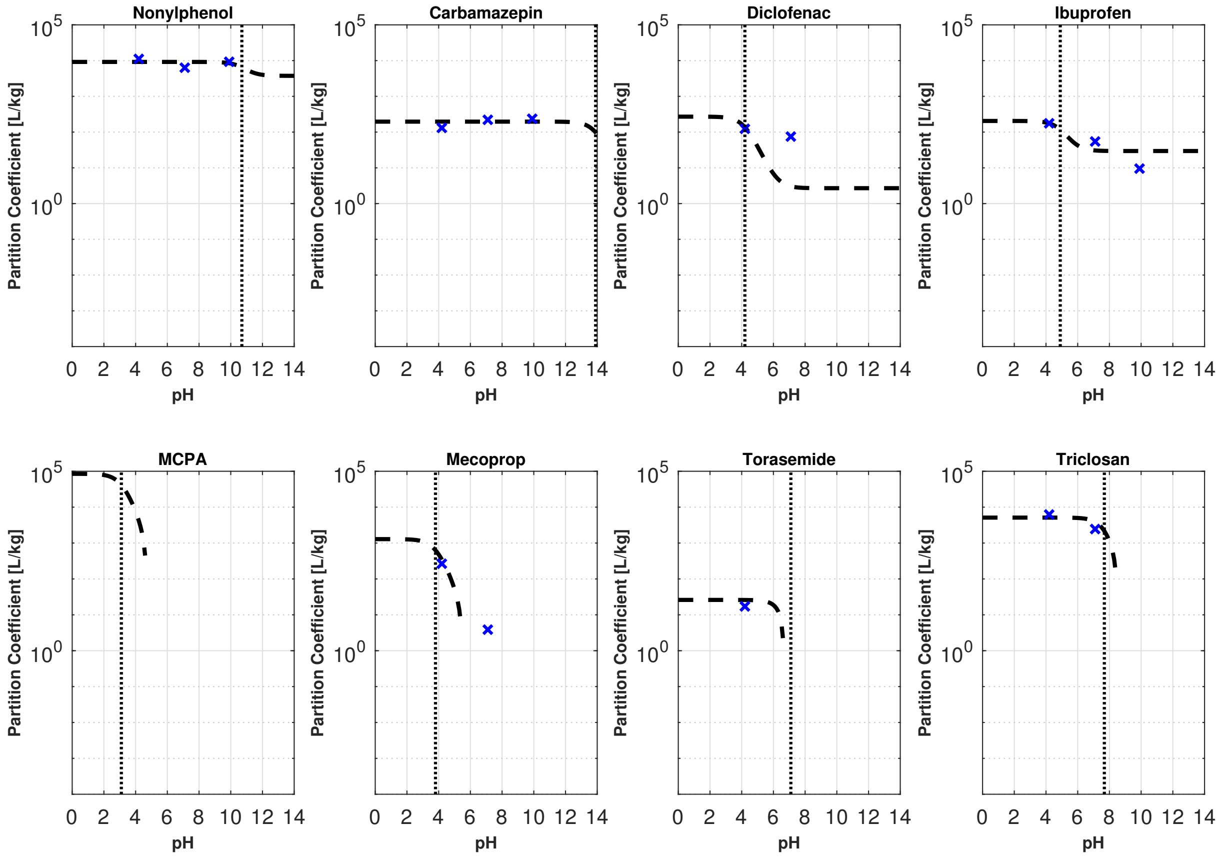

Figure S5: Comparison of measured D_P and theoretical partitioning for sorption of acids to polytstyrene illustrated by the blue crosses and dashed lines, respectively. The vertical dotted lines indicate the pK_a values. Due to the log-scale of the y-axis only positive values can be displayed.

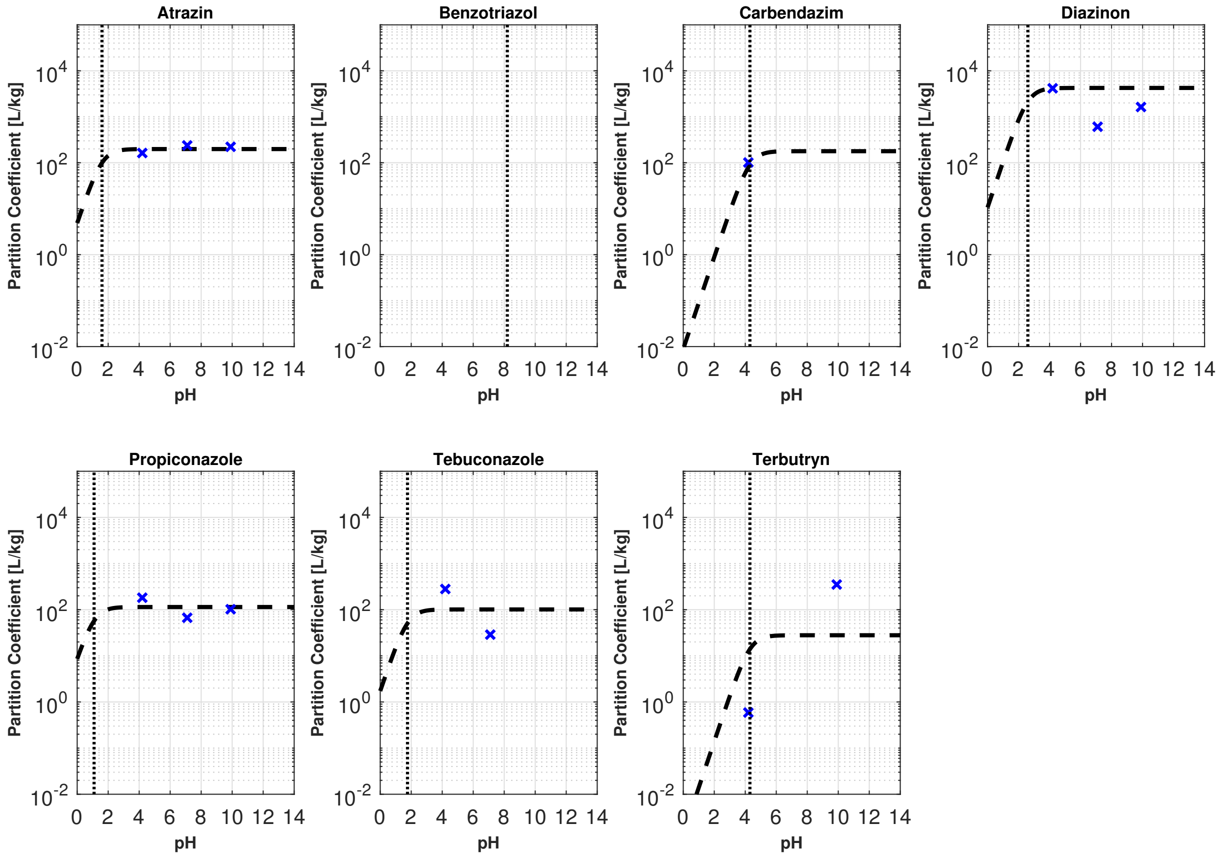

Figure S6: Comparison of measured D_P and theoretical partitioning for sorption of bases to polytstyrene illustrated by the blue crosses and dashed lines, respectively. The vertical dotted lines indicate the pK_a values. Due to the log-scale of the y-axis only positive values can be displayed.

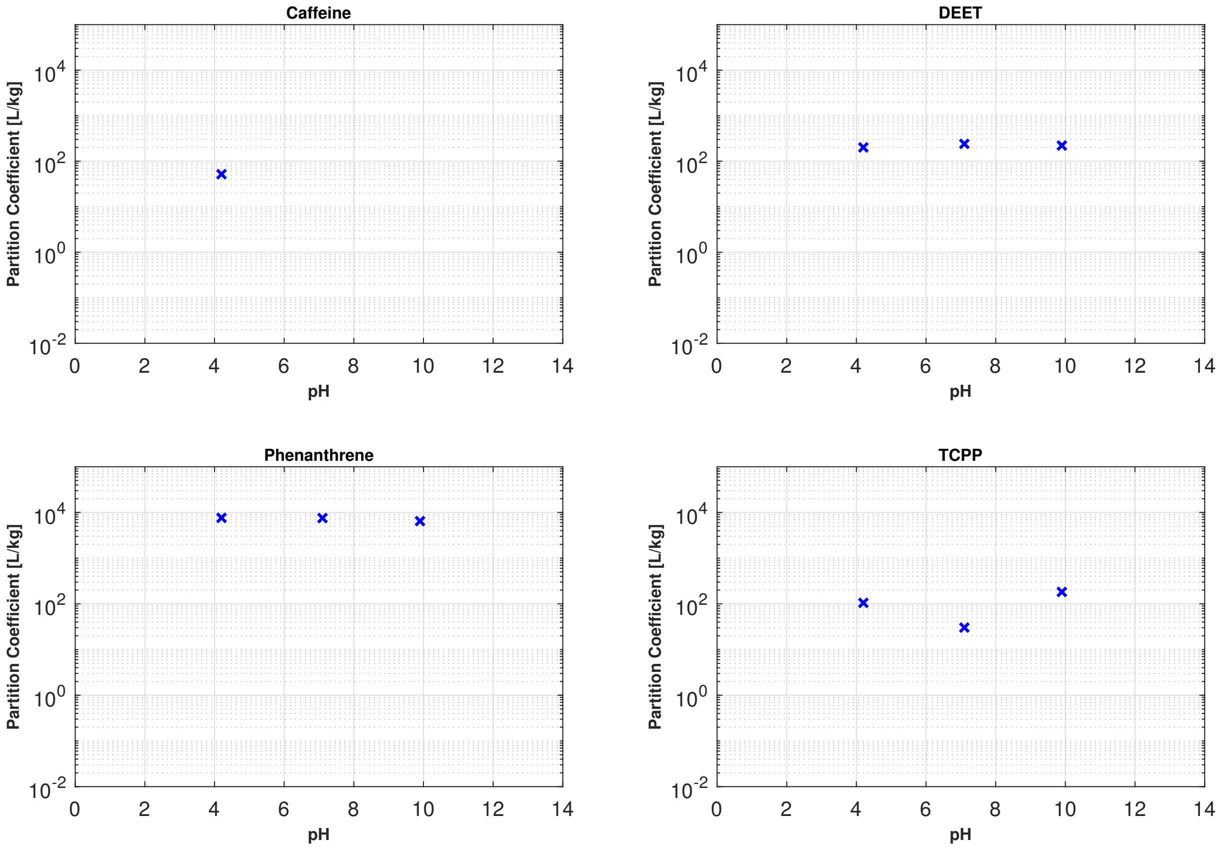

Figure S7: Measured D_P for sorption of neutrals to polytstyrene illustrated by the blue crosses. Due to the log-scale of the y-axis only positive values can be displayed.

S5 Isotherms

To evaluate sorption mechanisms for pollutants on PE and PS, we measured sorption isotherms. As sorbates phenanthrene (PAH), dibenzofuran (O-Heteroclylic), and dibenzothiophene (S-Heteroclylic) were used. The best fit for sorption on PE resulted in a linear isotherm whereas sorption on PS could best be described by a power function indicating nonlinear sorption mechanisms (Figure S8). R-squared values for all six isotherms were >0.98. The exponents of the fitted power functions were in the range of $0.75-0.78$. N_2-BET measurements were used to determine surface area and pore volumes of the different plastic particles. While PE is non-porous and had a surface area of 0.098 m^2g^{-1} (the applied method was not able to determine a pore volume), PS can be characterized as a porous material with a surface area of 0.6518 m^2g^{-1} and a pore volume of 0.0032 cm^3g^{-1}. Thus, different sorption isotherms can be explained by different involved mechanisms since pore-filling mechanisms may play a role in partitioning to PS.

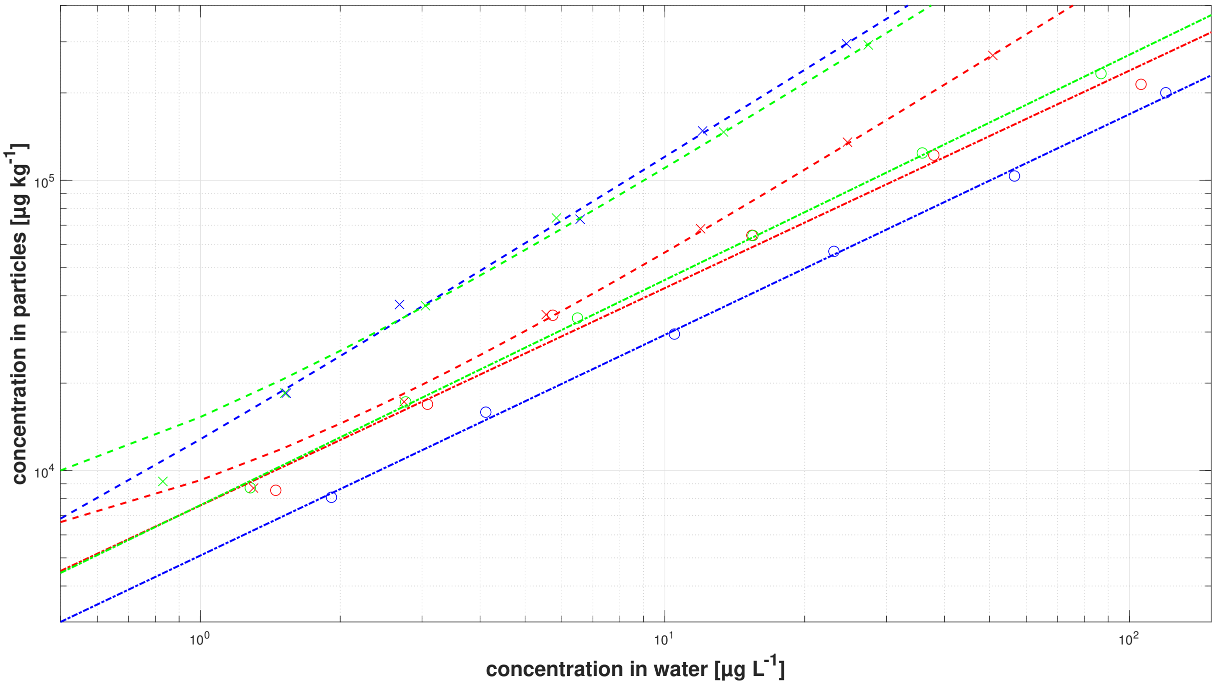

Figure S8: Sorption isotherms for phenanthrene (blue), dibenzofuran (red), and dibenzothiophene (green). Both axes scaled logarithmic. Crosses and circles show sorption to PE and PS, respectively.
